# In Vitro Insights into the Antifungal, Prebiotic, and Cytotoxic Potential of Tomato Plant Waste

**DOI:** 10.3390/nu17223616

**Published:** 2025-11-20

**Authors:** Simona Marcu Spinu, Mihaela Dragoi Cudalbeanu, Carmen Laura Cimpeanu, Nikola Major, Elwira Sieniawska, Krzysztof Kamil Wojtanowski, Ionela Avram, Diana Pelinescu, Alina Ortan, Narcisa Elena Babeanu

**Affiliations:** 1 Faculty of Land Reclamation and Environmental Engineering, University of Agronomic Sciences and Veterinary Medicine of Bucharest, 59 Marasti Boulevard, 011464 Bucharest, Romania; simona.spinu@fifim.ro (S.M.S.); mihaela.dragoi@fifim.ro (M.D.C.); alina.ortan@fifim.ro (A.O.); 2 Institute of Agriculture and Tourism, 52440 Poreč, Croatia; nikola@iptpo.hr; 3 Department of Natural Products Chemistry, Medical University of Lublin, 20-093 Lublin, Poland; esieniawska@pharmacognosy.org; 4 Department of Pharmacognosy with Medicinal Plants Garden, Medical University of Lublin, 20-093 Lublin, Poland; krzysztof.wojtanowski@umlub.edu.pl; 5 Department of Genetics, University of Bucharest, 1-3 Aleea Portocalelor, 060101 Bucharest, Romania; ionela.avram@unibuc.ro; 6 Faculty of Biotechnologies, University of Agronomic Sciences and Veterinary Medicine of Bucharest, 59 Marasti Boulevard, 011464 Bucharest, Romania; narcisa.babeanu@usamv.ro

**Keywords:** leaves waste, soluble sugars, glycoalkaloids, GC-MS, FTIR, *Candida* species, antifungal effect, prebiotic effect, cytotoxicity effect, health approach

## Abstract

Background/Objectives: This study aims to screen the extracts of tomato plant waste (aerial parts—mixture of leaves, stems, and bunches resulting from tomato crop maintenance, and axillary shoots—resulting from pruning practices) and evaluate their antifungal, prebiotic, and cytotoxic effects. Methods: A phytochemical profiling was performed to analyze volatile and semi-volatile compounds by GC-MS, functional groups by FTIR, soluble sugars by HPLC-RI, and glycoalkaloids by LC-MS/MS. Tomato plant waste extracts were further tested in vitro, and their biological effects were assessed with probiotic microorganisms (*Enterococcus faecium* ATCC 19434, *Enterococcus faecium* VL43, *Lactobacillus plantarum* ATCC 8014, and *Lactobacillus plantarum* GM3) to determine their prebiotic-like properties, particularly after demonstrating strong antifungal activity against several *Candida* species, such as *Candida albicans* ATCC 10231, *Candida parapsilosis* ATCC 22019, *Candida glabrata* ATCC 64677, and *Candida auris* 6328. The extracts were also evaluated for the cytotoxic effect against HEP-G2, HeLa, and HT-29 cell lines, while cytotoxicity assays confirmed no significant effects on the normal HEK-293 cell line compared to the control. Results: The in vitro antimicrobial activity and prebiotic-like substrate assay proved the difference between extract effects against *Candida* species (*C. glabrata*—MIC 125 µg/mL) and, respectively, the influence on *Lactobacillus* strains growth (up to a 1.6-fold increase in OD_600_). Furthermore, they exhibited selective cytotoxicity against HEP-G2, HeLa, and HT-29 cancer cell lines, while showing no significant toxicity on normal HEK-293 cells. Conclusions: Overall, this research highlights tomato axillary shoots as a sustainable source of bioactive compounds, with potential applications in developing natural, plant-based prebiotic products that exhibit antifungal and antitumor activity. This research focuses on developing natural, plant-based prebiotic products with antifungal and cytotoxic effects.

## 1. Introduction

Since plant materials represent one of the most abundant and accessible sources of prebiotics, current strategies for their utilization involve the recovery of these compounds from agro-industrial waste. These wastes are generated in large quantities annually by agricultural and related industries, and their use for various purposes would be highly beneficial for both the environment and the economy. If not properly managed, they represent a source of pollution, and their handling, from transportation to storage, incurs significant costs. Therefore, their valorization would help reduce both environmental pollution and economic burden [1,2,3].

At the same time, since agro-industrial waste originates largely from plants, it is rich in numerous bioactive compounds. Tomato processing by-products, for example, consist primarily of tomato seeds, peels, and residual vascular tissue. As a result, they are very rich in various phenolic compounds, β-carotene, lycopene, and amino acids with bioactive properties. Specifically, they are abundant in phenolic compounds and flavonoids, which contribute to their antioxidant and antifungal activity [4,5,6].

A little-explored agro-industrial waste of tomatoes is represented by tomato suckers, also referred to as axillary shoots or laterals, which arise in the leaf axil, between the main stem and a leaf. If left unpruned, these axillary shoots can develop into vigorous secondary stems with their own branches, flowers, and fruits, which then directly compete with the main stem for essential nutrients, water, and sunlight; as a consequence, they weaken the primary plant and are generally regarded as an agricultural waste [7].

The exploration of this waste is of particular interest, as it represents a miniature version of the mature plant and, therefore, holds the potential to benefit from the bioactive components typically present in a fully developed plant. This suggests that such waste could serve as reservoirs of valuable bioactive substances. These compounds, normally found in mature plants, could be isolated from waste without the need to sacrifice the mature plant itself, thus increasing overall yield. Moreover, the waste exhibits a significant growth rate, further enhancing its potential utility [8,9,10,11]. Among the valuable bioactive compounds that this plant waste may contain is tomatine, a glycoalkaloid that accumulates in every organ of the tomato plant. Tomatine has demonstrated a broad spectrum of beneficial biological properties, including antioxidant, anti-inflammatory, antibiotic, and antifungal activities [12,13]. In addition to tomatine, the waste may also be rich in phenolic compounds and flavonoids, both known for their potent antioxidant activity. Furthermore, carotenoids such as lycopene and β-carotene, typically found in whole Heinz hybrid tomatoes and their by-products, are also likely to be present. These compounds are well recognized for their health-promoting effects. The waste may also contain essential vitamins, including vitamin A, vitamin C, and vitamin E, contributing further to its bioactive profile [14,15]. At the same time, it could also contain alkaloids, since tomato plant by-products are identified as promising sources of alkaloids in general. Additionally, it may contain a wide range of chitinases, with 43 different isoforms identified to date. These enzymes are expressed during various developmental stages of the plant and play a key role in its defense against phytopathogens [16]. Recent studies provided data regarding the prebiotic effect of tomato extracts by in vitro or ex vivo experiments, but the mechanisms are still uncertain. Emerging directions include integration of metabolomics to determine biotransformation of bioactive compounds from tomato waste by the gut microbiota and fermentation strategies in order to obtain efficient, standardized, and safe prebiotic ingredients [17].

While the prebiotic potential of tomato fruit processing by-products like seeds and peels is increasingly recognized, research on the aerial plant waste—leaves, stems, and especially axillary shoots—remains limited. Existing studies on these green residues have primarily described their composition and general bioactivity, but a critical gap exists in systematically evaluating their prebiotic effects alongside other relevant biological properties. Specifically, the combined assessment of their antifungal efficacy against clinically relevant *Candida* strains and their selective cytotoxicity, within the context of a potential prebiotic application, has not been explored. This study addresses this gap by providing a comprehensive in vitro investigation of tomato aerial waste, not only profiling its rich phytochemical composition but also quantitatively demonstrating its trifunctional potential as a source of natural prebiotic, antifungal, and antitumoral agents.

This study aims to evaluate the antimicrobial, prebiotic, and cytotoxic effects of tomato aerial parts and axillary shoots, with a view to harnessing their potential application across various industries, particularly the food and medical sectors. This approach aligns with the principles of a circular and sustainable economy, promoting the efficient use of agricultural by-products. The hypothesis regarding the prebiotic potential of tomato aerial parts mixture and tomato axillary shoots is supported by their biochemical composition, notably their content of polysaccharides, dietary fiber, and phenolic compounds, all known contributors to gut health and microbiome modulation [18]. In this study, in vitro tests were performed to evaluate the prebiotic potential of tomato waste extracts, aiming to identify bioactive compounds that could be further exploited in human health applications.

## 2. Materials and Methods

### 2.1. Developing the Novel Prebiotic Formulation of Tomato Plant Waste Extracts

The tomato plant waste was collected from the Research Greenhouse at the University of Agronomic Sciences and Veterinary Medicine of Bucharest, Bucharest, Romania. Tomato plant waste included aerial parts, a mixture of leaves, stems, and bunches from tomato crop maintenance, as well as axillary shoots resulting from pruning practices of *Lycopersicon esculentum* hybrid Cheramy RZ F1.

The extraction and optimization of tomato plant waste extracts were previously reported [19]. The extraction was conducted using ethanol as the solvent and three extraction methods: microwave-assisted extraction, ultrasound-assisted extraction, and ultrasound-assisted extraction followed by microwave-assisted extraction (cascade extraction). The optimization was conducted using a Box–Behnken design combined with Response Surface Methodology. A total of 17 experimental runs were carried out, including five centroid points, three levels, and three independent variables (solvent concentration, solvent-to-plant ratio, and temperature). After all, eight tomato plant waste extracts optimized were used in this study, as described in Table 1.

Aimed at phytochemical profiles, tomato plant extracts were prepared separately for each assay. For example, for the volatile and semi-volatile (GC-MS) and functional groups (FTIR) analyses, extracts were dissolved in methanol (Sigma-Aldrich, St. Louis, MO, USA) to obtain a 10 mg/mL solution. Regarding soluble sugar (HPLC-RI) and glycoalkaloids (LC-MS/MS) analyses, extracts were dissolved in 80% methanol to obtain a 1 mg/mL solution and a 10 mg/mL solution, respectively. After all, the extracts were filtered using 0.45 μm polytetrafluoroethylene (PTFE) microfilters (Corning, New York, NY, USA). Concerning in vitro biological assays, the tomato plant waste extracts were dissolved in dimethyl sulfoxide (DMSO) (Sigma-Aldrich, St. Louis, MO, USA) to obtain a 40 mg/mL stock solution. Sterilization of the extracts was performed using a 0.22 μm polytetrafluorethylene (PTFE) microfilter (Corning, New York, NY, USA).

### 2.2. Phytochemical Profiles of the Tomato Plant Waste Extracts

#### 2.2.1. Assessment of GC-MS Volatile and Semi-Volatile Profile

Gas Chromatography coupled with Mass Spectrometry (GC-MS) analysis of the tomato plant waste extracts (methanolic extracts at a concentration of 10 mg/mL) was performed after the optimization of the extraction parameters for the volatile and semi-volatile compounds. Chromatographic separation and mass spectrometry detection were carried out using a Shimadzu GC 2010 Plus chromatograph coupled with a Shimadzu QP 2010 Ultra mass spectrometer (Shimadzu, Kyoto, Japan). The entire system was equipped with an AOC-20I auto-injector and an AOC-20S autosampler (Shimadzu, Kyoto, Japan). ZB-5MS capillary column (30 m) with a silica film thickness of 0.25 µm and an internal diameter of 0.25 mm (Phenomenex, Torrance, CA, USA) was used. The initial oven temperature was 50 °C, with 3 min holding time, and then increased to 250 °C at 5 °C/min and held for 15 min at 250 °C. Helium, used as a carrier gas (1 mL/min), was maintained at 280 °C during GC injection (1 µL) in split mode (1:20). The electron impact ion source operated at 70 eV energy. The scan rate and range were 0.2 s/scan and 40–500 amu, respectively. The temperature of the ion trap was set at 220 °C, while the temperature of the MS injection and interface was 250 °C. The relative retention indices (RRI) of compounds present in the samples were calculated with respect to the homologous series of n-alkanes (C6–C30) analyzed under identical conditions. The identification of metabolites was supported by the NIST 2011 spectral library and available literature data [20].

#### 2.2.2. Assessment of FTIR Phytochemical Profile

Fourier-transform infrared (FTIR) spectroscopy was employed to identify phytochemical functional groups in the tomato plant waste extracts, using a Tensor 27 spectrometer (Bruker, Bruker Optics GmbH, Ettlingen, Germany) equipped with a diamond crystal attenuated total reflectance (ATR) accessory (Platinum ATR). Using this accessory, tomato plant waste extracts (methanolic extracts at a concentration of 10 mg/mL) were analyzed directly to obtain characteristic absorption spectra in the wavenumber range of 4000–400 cm^−1^. The spectral acquisition resolution was 4 cm^−1^, and each spectrum represented the average of 32 scans.

#### 2.2.3. Assessment of the HPLC Soluble Sugar Profile

The analysis of soluble sugars, including sucrose, glucose, fructose, and fructooligosaccharides (quantified as inulin), was performed using a Shimadzu High-Performance Liquid Chromatography system (Shimadzu, Kyoto, Japan) containing a computer-controlled CBM-40 unit, a DGU-405 degasser, an LC-20Ai pump, a SIL-20AC autosampler, a CTO-40S column oven for temperature optimization, and an RID-20A refractive index detector. Intended for compound separation, 30 µL of each extract under investigation was injected on a 300 × 8 mm Ca^2+^ column, with a 9 µm particle size (Repromer Ca, Dr. Maisch, Ammerbuch-Entringen, Germany). The column temperature was maintained at 80 °C, and ultrapure water served as the mobile phase at a constant flow rate of 0.6 mL/min for a total run time of 40 min. The tomato plant waste extracts were dissolved in hydroalcoholic solvent (80% methanol) to obtain a concentration of 1 mg/mL.

#### 2.2.4. Assessment of the LC-MS/MS Glycoalkaloids Profile

Chromatographic separation of the glycoalkaloids present in the tomato plant waste extracts was performed on a C18 Gemini column (3 μm, 110 Å, 100 × 2 mm, with TMS end-capping) equipped with a guard column (Phenomenex Inc., Torrance, CA, USA). The system used was an Agilent 1260 Infinity HPLC (Agilent Technologies, Santa Clara, CA, USA). Glycoalkaloids were eluted with a solvent mixture of water containing 0.1% formic acid (A) and acetonitrile with 0.1% formic acid (B). The gradient program was: 0–60% B over 45 min, then 60–95% B for 1 min, followed by 95% B for 9 min, at a flow rate of 0.2 mL/min. A 10 μL injection of each extract (80% methanolic extracts at a concentration of 10 mg/mL) was carried out at 20 °C. Detection was performed using an Agilent 6530B QTOF system (Agilent Technologies, Santa Clara, CA, USA) in positive ion mode, with collision energies of 10 and 30 eV. The scan range was 50–1700 *m*/*z* with two spectra acquired per second. Additional parameters included: drying gas temperature at 275 °C, flow at 10 L/min; sheath gas temperature at 325 °C, flow at 12 L/min; nebulizer pressure at 35 psi; capillary voltage (+) at 4000 V; skimmer voltage at 65 V; and fragmentor voltage at 140 V. Glycoalkaloids identification was tentative, based on accurate masses, fragmentation patterns, and literature sources.

### 2.3. In Vitro Biological Effects of the Tomato Plant Waste Extracts

#### 2.3.1. Assessment of the Antifungal Effect Using the Disk Diffusion Assay

The antifungal effects of tomato plant waste extracts were assessed using the disk diffusion assay. Six yeast strains, including *Candida albicans* ATCC 10231, *Candida albicans* CMGBy 18, *Candida parapsilosis* ATCC 22019, *Candida glabrata* ATCC 64677, *Candida auris* DSM21092, and *Candida auris* 6328, were chosen as test strains. Reference strains were obtained from accredited international culture collections, while the other strains originated from the MICROGEN Center microbial collection at the Faculty of Biology, University of Bucharest, which is part of the Microbial Resource Research Infrastructure (MIRRI). Yeast Glucose Chloramphenicol (YCG) Agar (Liofilchem, Roseto degli Abruzzi, TE, Italy) was used as a culture medium to measure the inhibition zones. Fresh microbial cultures were made to a 0.5 McFarland density. Then, the yeast suspension was swabbed onto YCG agar plates. Sterile discs (Oxoid™ Antimicrobial Susceptibility Individual disc—Thermo Fisher Scientific, Pittsburgh, PA, USA) and 10 μL of tomato plant waste extract (40 mg/mL) were placed on the plates. The plates were incubated at 37 °C for 24 h, and the zones of inhibition were measured afterward.

#### 2.3.2. Assessment of the Antifungal Effect Using the Microdilution Assay

The antifungal effects of tomato plant waste extracts were evaluated using a broth microdilution assay, following the Clinical and Laboratory Standards Institute (CLSI) M27 standard recommendations. Four yeast strains, including *Candida albicans* ATCC 10231, *Candida parapsilosis* ATCC 22019, *Candida glabrata* ATCC 64677, and *Candida auris* 6328, were selected for testing. Stock solutions (40 mg/mL) of the extracts were diluted in Phosphate-Buffered Saline (PBS) at a 1:10 ratio to obtain an intermediate solution of 4 mg/mL. This intermediate solution was further diluted in broth medium RPMI-1640 (Gibco, Billings, MT, USA) to reach a final concentration of 2 mg/mL. The extracts (100 μL) were serially diluted in a 1:1 ratio by volume, and then 10^3^ cells/mL of *Candida* were added to each well. The plates were incubated for 24 h at 37 °C, and absorbance was measured at λ660 using a microplate reader (Synergy™ HTX Multi-Mode Microplate Reader, Biotek, Winooski, VT, USA). The results were expressed as minimum inhibitory concentration (MIC). The MIC values are interpreted as the lowest concentration of tomato plant waste extracts that inhibit noticeable growth for *Candida* species, compared to the growth control without extracts [21]. Fluconazole was used as a drug control.

#### 2.3.3. Assessment of the Prebiotic Effect Using the Growth Curve Kinetics Assay

Four probiotic microorganisms (*Enterococcus faecium* ATCC 19434, *Enterococcus faecium* VL43, *Lactobacillus plantarum* ATCC 8014, and *Lactobacillus plantarum* GM3) were tested to assess the prebiotic effects of tomato plant waste extracts. Reference probiotics were obtained from accredited international culture collections, while the other probiotics originated from the MICROGEN Center microbial collection at the Faculty of Biology, University of Bucharest, which is part of the Microbial Resource Research Infrastructure (MIRRI). Probiotic microorganisms were analyzed without glucose as a carbon source. The probiotic microorganisms were cultured in the Man–Rogosa–Sharpe (MRS, Liofilchem, Roseto degli Abruzzi, TE, Italy) agar medium at 37 °C for 24 h. Stock solutions (40 mg/mL) of the extracts were diluted in PBS at a 1:10 ratio to obtain an intermediate solution of 4 mg/mL. This intermediate solution was further diluted in broth MRS media to reach a final concentration of 2 mg/mL. A 100 μL of extracts and 100 μL of inoculum (0.5 McFarland) were added to the microplate, and the microplate was incubated at 37 °C for another 48 h. The absorbance was measured at λ600 every 30 min using a microplate reader (Synergy™ HTX Multi-Mode Microplate Reader, Biotek, Winooski, VT, USA). The prebiotic effect refers to tomato plant waste extracts that beneficially affect probiotic microorganisms by selectively stimulating growth compared to the growth control without extracts [22].

#### 2.3.4. Assessment of the Cytotoxicity Effect Using In Vitro Assay

The cytotoxicity effect of tomato plant waste extracts was evaluated using the MTT (3-(4,5-dimethylthiazol-2-yl)-2,5-diphenyltetrazolium bromide) tetrazolium reduction assay on HEK-293 (embryonic kidney), HEP-G2 (liver tumor), HeLa (cervical tumor), and HT-29 (colon tumor) cell lines (CLS Cell Lines Service GmbH, Eppelheim, Deutschland). The HEK-293 cell line was cultivated in Fibroblast Medium (FM) culture medium (Innoprot, Derio, Spain), and the HEP-G2, HeLa, and HT-29 cell lines were cultivated in Dulbecco’s Modified Eagle Medium (DMEM) culture medium (Sigma-Aldrich, St. Louis, MO, USA). The culture medium was supplemented with 10% Fetal Bovine Serum (FBS) (Sigma-Aldrich, St. Louis, MO, USA) and 1% penicillin-streptomycin (Sigma-Aldrich, St. Louis, MO, USA). All the cell lines were exposed to tomato plant waste extracts and incubated in 5% CO_2_ at 37 °C for 24 h. The stock solutions (40 mg/mL) of extracts were diluted in Phosphate-Buffered Saline (PBS) to 1:10 to obtain an intermediate solution of 4 mg/mL. The intermediate solution was further diluted to the required concentrations (200 and 400 μg/mL) using culture medium. H_2_O_2_ addition to cells was used as a positive control, and 1% DMSO in PBS was used as a negative control. After incubation, the supernatant was removed, and the cells were treated with 100 µg/mL MTT. The plates were then incubated at 37 °C for 4 h. The purple formazan formed was dissolved in 100 μL of DMSO, and the optical density was measured at λ570 nm using a Synergy HTX Multi-Mode Microplate Reader (Biotek, Winooski, VT, USA). The cytotoxic effect of the tomato plant waste extracts was reported as percentage inhibition compared to the positive and negative controls.

### 2.4. Statistical Analysis

Experiments were conducted independently in triplicate (*n* = 3), and the results are shown as means ± standard deviation. To assess statistical significance, a One-way ANOVA was performed using GraphPad Prism Software version 10.5.0 (Boston, MA, USA). Following this, Tukey’s test was used to determine significant differences between the means, with a significance threshold set at *p* < 0.05.

## 3. Results

### 3.1. Phytochemical Profiles of the Tomato Plant Waste Extracts

#### 3.1.1. GC-MS Volatile and Semi-Volatile Profile

The volatile and semi-volatile compounds present in the tomato plant waste (TPW) extracts were analyzed using GC-MS analysis. Table 2 presents these compounds along with their retention time (RT), relative retention index (RRI), molecular weight (MW), and relative area (%). Across the TPW extracts analyzed, a total of 16 compounds were identified. Among these, eight belong to the class of esters, three to fatty amides, and two to hydrocarbons. In addition, one aromatic ketone, one isoprenoid alcohol, and one fatty alcohol were identified.

Among the most abundant compounds identified in all analyzed TPW extracts were phytol and the methyl esters of palmitic, linoleic, and linolenic acids. Notably, phytol was found in considerably higher abundance in UAE (Usas—23.66% and USap—29.70%) compared to MAE (13.46% MWas and 0.90% MWap). In the case of methyl esters of palmitic, linoleic, and linolenic acids, these esters were found in considerably higher abundance in MAE. The increasing order of relative areas was identical for the types of plant material (aerial parts—ap and axillary shoots—as), with the highest values recorded in MWas extract and the lowest values in MWap extract.

Several compounds, including 3′,5′-dimethoxyacetophenone, hexadecatrienoic acid methyl ester, and octadecatrienol, were identified exclusively in TPW extracts obtained by UAE. In contrast, methyl 18-methylnonadecanoate and linoleic acid ethyl ester were detected exclusively in extracts obtained through MAE. Distinct chemical signatures were also observed within individual TPW extracts. Specifically, the TPWap extracts contained hexyl-methylcyclopentane, 3′,5′-dimethoxyacetophenone, and nonadecanamide as unique constituents. Regarding the TPWas extracts, the following compounds were exclusively identified: methyl 18-methylnonadecanoate, linoleic acid ethyl ester, and 2-methylhexacosane. Only through cascade extractions (CAS2) was nonadecanamide exclusively identified.

The GC-MS analysis uncovers clear trends in the composition of TPW extracts obtained through various extraction methods (MAE, UAE, CAS1, CAS2) and offers valuable insights into extraction efficiency and compound stability. For instance, phytol showed significantly higher relative abundance in UAE extracts (23.66–29.70%) compared to MAE (0.90–13.46%), indicating that ultrasonic waves effectively release this diterpenoid alcohol while reducing thermal degradation. Conversely, palmitic acid methyl ester was more prevalent in MAE extracts (up to 16.05% in MWas extract) than in UAE extracts, suggesting that MAE favors the release of certain fatty acid esters, potentially due to better cell wall disruption and localized heating.

Other trends include octadecatrienoic acid methyl ester, which appeared more prominently in MAE extracts (up to 22.23% in MWas extract) compared to UAE, highlighting method-specific selectivity toward polyunsaturated fatty acids. Similarly, octadecenamide was more effectively extracted using MAE, while UAE preferred more thermolabile or less polar compounds such as phytol. These findings indicate that the choice of extraction method influences not only the overall yield but also the relative composition of volatile and semi-volatile compounds. MAE tends to favor the extraction of higher molecular weight fatty acid derivatives and amides, likely due to enhanced matrix heating. In contrast, the UAE efficiently isolates less polar, thermostable compounds without significant degradation.

These findings highlight the strong influence of extraction methodology (UAE, MAE, and CAS) on the chemical composition of TPW extracts, underlining the method-dependent recovery of specific bioactive or structurally distinctive metabolites.

#### 3.1.2. FTIR Phytochemical Profile

**Figure 1:**
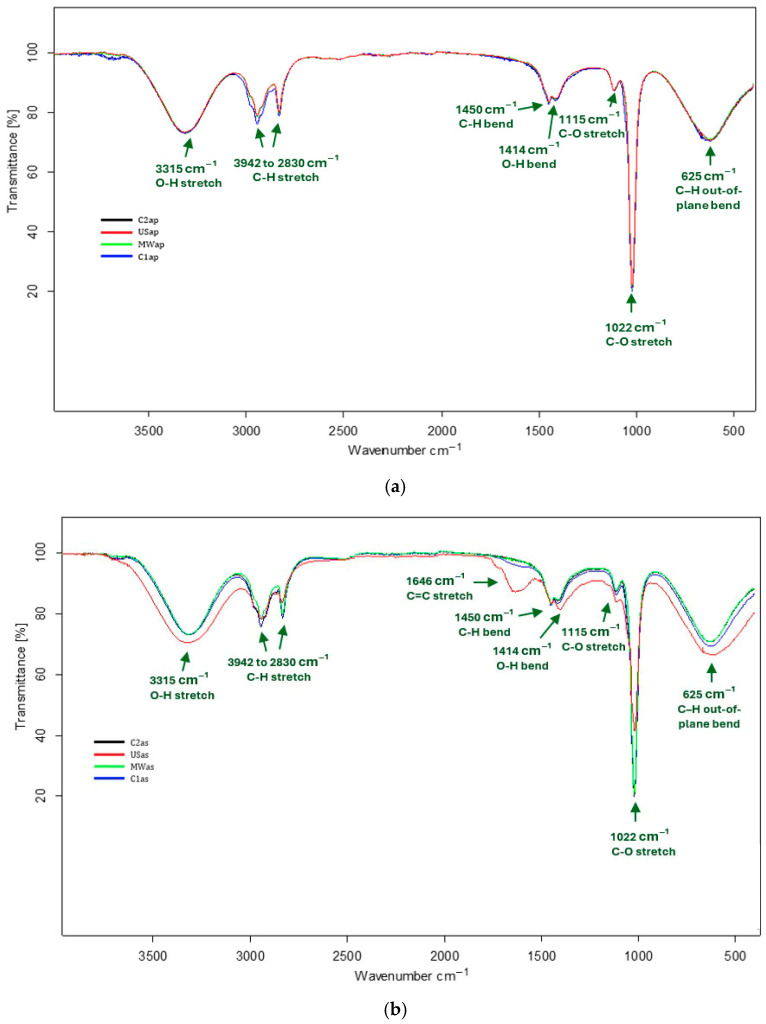
FTIR spectra associated with TPW extracts—(**a**) TPWap and (**b**) TPWas.

The FTIR profile analysis of the TPW extracts was conducted to identify the functional groups of phytochemical compounds based on the absorption band values in the infrared region. The FTIR spectra recorded for TPWap extracts are presented in Figure 1a, while those corresponding to the TPWas are shown in Figure 1b, for the four extraction methods applied (MAE, UAE, CAS1, CAS2). This arrangement enables a direct comparison of the extraction techniques through spectral superposition. The superimposed spectra revealed absorption bands that were common or closely aligned across the four extraction methods, in both TPWap and TPWas extracts. The similarity in the wavenumber ranges (617–3706 cm^−1^) indicates the occurrence of comparable functional groups in all extracts.

Regarding the TPWap extracts, the main absorption bands were identified as follows: 3315 cm^−1^, corresponding to the stretching vibrations of the O–H bonds in polysaccharides or phenolic compounds [23,24]; 2942 and 2831 cm^−1^, associated with the stretching vibrations of the C–H bonds in alkanes [25]; 1450 cm^−1^, attributed to the bending vibrations of C–H bonds in alkanes [26]; 1414 cm^−1^, associated with the bending vibrations of O–H bonds in carboxylic acids; 1115 cm^−1^ and 1022 cm^−1^, related to the stretching vibrations of C–O bonds in alcohol groups of soluble sugars or phenolic compounds [27]; and 625 cm^−1^, attributed to the bending vibrations of the C–H out-of-plane in substituted aromatic rings [28].

In the case of TPWas extracts, the absorption bands were generally similar to those observed in the TPWap extracts, with the exception of a distinctive band at 1646 cm^−1^, which is assigned to the stretching vibrations of the C=C bonds in phenolic compounds, specifically flavonoids [29]. This band was markedly more intense in the USas extract, next to stretching vibrations of the O–H bonds and in bending vibrations of the C–H out-of-plane in substituted aromatic rings, although the USas extract shows lower intensity in the stretching vibrations of C–O bonds. These spectral features suggest a higher concentration of flavonoids and a reduced glucose content in the USas extract. By contrast, the MWas, C1as, and C2as extracts displayed fewer intensive flavonoid-associated bands alongside stronger C–O stretching signals, indicating a lower flavonoid concentration and a greater abundance of soluble sugar.

#### 3.1.3. HPLC Soluble Sugar Profile

**Figure 2:**
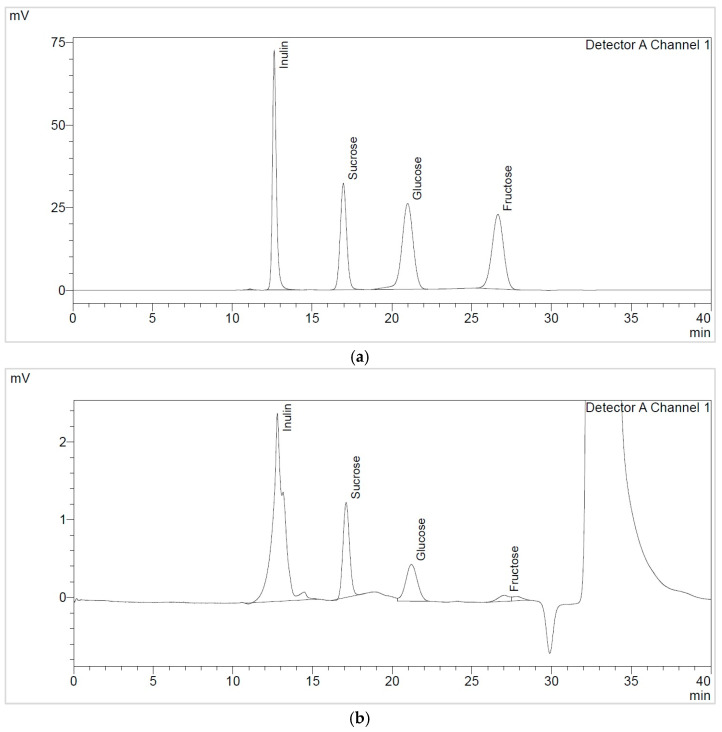
HPLC soluble sugars chromatogram of (**a**) reference standards and (**b**) MWap extract.

The soluble sugars identified in the TPW extracts, namely TPWap and TPWas, were sucrose, glucose, fructose, and fructooligosaccharides (FOS), quantified as inulin, as shown in Figure 2. Their concentrations are summarized in Table 3.

TPWap extracts exhibited higher sucrose compared to glucose. In the case of TPWas extracts, the concentration of soluble sugars, specifically sucrose, decreased by around 50%. Additionally, it was observed that the USas extract contains more fructose (26 ± 1 mg/g d.e.) than the other TPWas extracts, as well as the TPWap extracts. These aspects can also be visualized in the FTIR spectra; the USas extract showed a strong signal in the 950–600 cm^−1^ region, which can be attributed to simple sugars, such as fructose [30]. TPWap extracts contained higher amounts of sucrose and glucose compared to those TPWas extracts. The total soluble sugar content identified was higher in TPWap extracts compared to those in TPWas.

When comparing the extraction methods employed, MAE proved superior to UAE extraction in terms of total sugar content present in TPWap extracts. CAS2 extraction was more effective than CAS1 extraction, indicating a direct influence of extraction time on sugar yield. The total FOS content was also determined, ranging from 334 to 499 mg/g d.e.

#### 3.1.4. LC-MS/MS Glycoalkaloids Profile

Analyses using LC-MS/MS clearly confirmed the presence of glycoalkaloids in the TPW extracts. The glycoalkaloids were tentatively identified by comparing their spectral data to literature and analyzing fragmentation patterns. The four main glycoalkaloids found in TPW extracts were tomatidine, dehydrotomatine, α-tomatine, and acetoxytomatine. In addition to these glycoalkaloids, seven other α-tomatine derivatives were tentatively identified. Overall, 11 glycoalkaloids were identified in the TPW extracts (Table 4).

**Figure 3:**
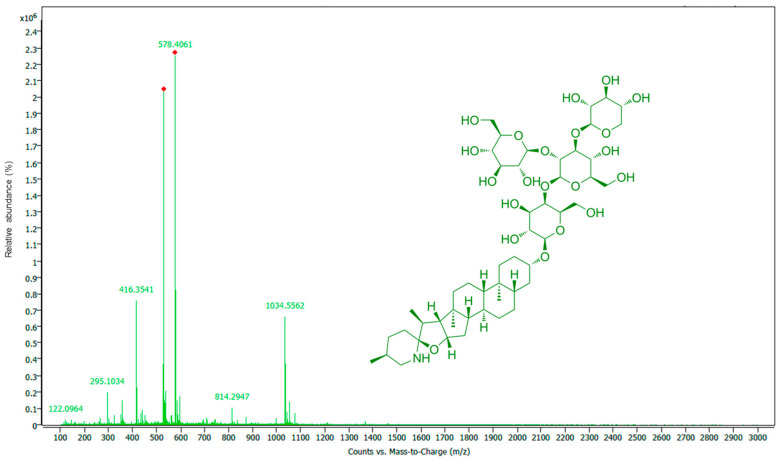
MS spectrum of TPW extract with α-tomatine structure.

The positive ion mass spectrum of α-tomatine is shown in Figure 3, with the six major ions assigned. In addition to the [M+H]^+^ ion (*m*/*z* 1034.5562), significant fragment ions at *m*/*z* 578.4061 and 416.3541 were also observed [36]. It is suggested that the secondary amine in tomatidine is readily protonated during the ionization process. The ions at *m*/*z* 578.4061 and 416.3541 correspond to [Tomatidine+Gal+H]^+^ and [Tomatidine+H]^+^, respectively. These fragments are primarily produced by the elimination of the Xyl-Glc(-Glc-) moiety and the complete sugar chain.

### 3.2. Positive In Vitro Biological Effects of the Tomato Plant Extracts

#### 3.2.1. Antifungal Effect Using Disk Diffusion Assay

The results of the antifungal effect of TPW extracts on the *Candida* species studied (*C. albicans* ATCC 10231, *C. albicans* CMGBy 18, *C. parapsilosis* ATCC 22019, *C. glabrata* ATCC 64677, *C. auris* DSM 21092, and *C. auris* 6328) are presented in Table 5. According to these results, the TPW extracts exert antifungal activity against all tested species. The highest inhibition activity was observed in all extracts tested against *C. glabrata* ATCC 64677, the most sensitive microorganism. Additionally, high inhibition activity was observed in *C. parapsilosis* ATCC 22019 when TPWas extracts were applied, compared to TPWap extracts.

Moderate inhibition activity of TPW extracts was observed against both *C. albicans* species. Therewith, MWas, C1as, and C2as presented high activity against *C. albicans* ATCC 10231, while USas showed higher activity against *C. albicans* ATCC 10231. Observing the differences between the antifungal activity of TPW extracts, regarding the types of plant material studied, higher inhibition activity can be observed in the case of TPWas extracts compared to TPWap extracts tested against *C. albicans* ATCC 10231, *C. parapsilosis* ATCC 22019, and *C. glabrata* ATCC 64677. No differences were observed in the antifungal effect of the TPW extracts against *C. auris* DSM 21092, *C. auris* 6328, and *C. albicans* CMGBy 18, regarding the types of plant material.

#### 3.2.2. Antifungal Effect Using the Microdilution Assay

The results of the antifungal effect using the microdilution assay are shown in Table 6 for each TPW extract tested against the four yeast species: *C. albicans* ATCC 10231, *C. parapsilosis* ATCC 22019, *C. glabrata* ATCC 64677, and *C. auris* 6328. These *Candida* species were more sensitive to TPW extracts than *C. albicans* CMGBy 18 and *C. auris* DSM 21092 (Table 5). Based on the results, a dose-dependent inhibition is generally observed. All TPW extracts showed inhibitory activity against *C. albicans* ATCC 10231, *C. parapsilosis* ATCC 22019, and *C. glabrata* ATCC 64677, with higher effects in the case of TPWas extracts. Also, a lower effect was observed for all TPW extracts against *C. auris* 6328. However, variations were observed depending on the extraction method, with cascade extractions (CAS1 and CAS2) being more efficient than MAE and UAE for TPWap extracts in the case of *C. albicans* ATCC 10231. For example, testing against C. glabrata ATCC 64677, which was the most sensitive strain, showed that the TPW extracts were significantly more potent, with the lowest minimum inhibitory concentration (MIC) values ranging from 125 µg/mL (MWap, MWas, Usas, C1ap, C1as, and C2as) to 250 µg/mL (USap and C2ap). Testing against *C. parapsilosis* ATCC 22019 showed that the three most promising TPW extracts, with the lowest MIC values, were MWas, Usas, and C2as (250 µg/mL), highlighting the variation based on the types of plant material used (axillary shoots vs. aerial parts). In comparison, the results obtained for *C. auris* 6328 showed that the microbial strain exhibits high resistance to these extracts, with MIC values of 1000 µg/mL.

#### 3.2.3. Prebiotic Effect Using the Growth Curve Kinetics Assay

**Figure 4:**
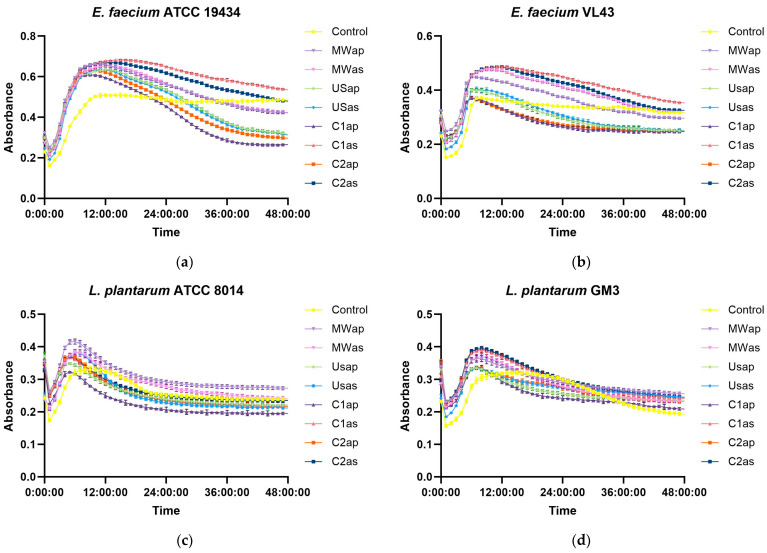
Prebiotic effect of TPW extracts—substrate used without glucose as a carbon source, recorded at OD_600_—(**a**) *E. faecium* ATCC 19434; (**b**) *E. faecium* VL43; (**c**) *L. plantarum* ATCC 8014; (**d**) *L. plantarum* GM3.

The prebiotic effects of TPW extracts, used as substrates, were explored by assessing their ability to stimulate the growth of four specific lactic acid bacteria, including *E. faecium* ATCC 19434, *E. faecium* VL43, *L. plantarum* ATCC 8014, and *L. plantarum* GM3, without glucose as a carbon source. Because certain lactic acid bacteria exhibited poor growth on MRS medium lacking glucose, this study selected only those probiotic strains capable of sustaining growth and proliferation for subsequent co-cultivation on MRS with TPW extracts. However, it was observed that all TPW extracts exhibited excellent prebiotic effects, promoting the growth of all tested probiotic microorganisms compared to the control (Figure 4). These findings could be attributed to the high carbohydrates (see Section 3.2.2 and Section 3.2.3) and phenolic compounds [19] present in the TPW extracts, which enhance the growth of beneficial probiotic microorganisms.

The growth curves of bacterial strains cultivated with TPW extracts exhibited a similar profile, characterized by a pronounced exponential phase, whereas the controls without extracts showed reduced growth and an extended stationary phase. All strains reached their maximum growth at approximately 8 h, followed by a decline due to carbon source depletion. In the absence of glucose, sugars from the TPW extracts stimulated bacterial proliferation, as illustrated in Figure 3. In *E. faecium*, two extracts (C1as and C2as) resulted in higher growth values than the control at 48 h, while in *L. plantarum* GM3, all extracts enhanced growth.

#### 3.2.4. Cytotoxicity Effect Using In Vitro Assay

**Figure 5:**
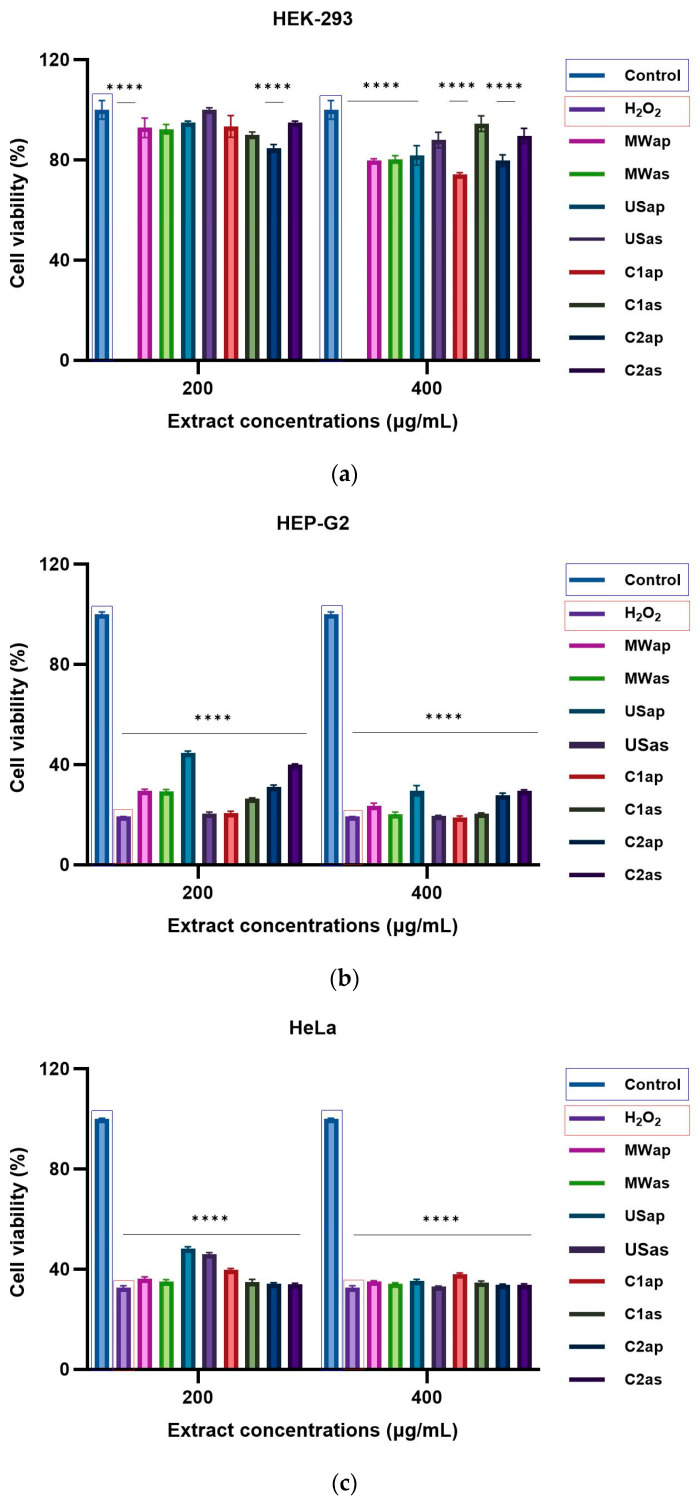

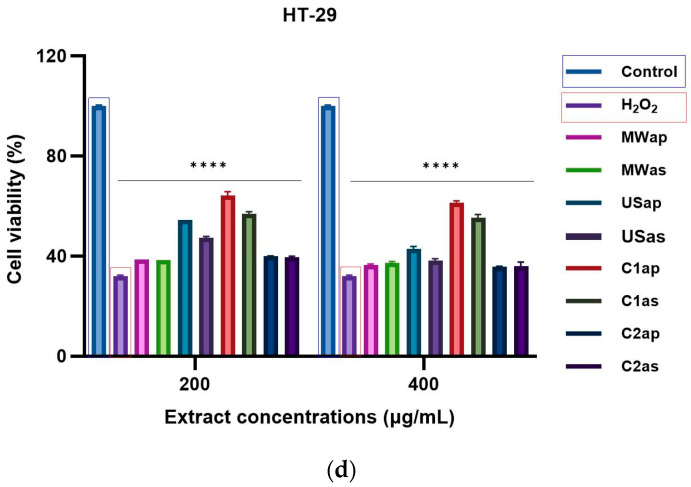
Cytotoxicity effect in cells treated for 24 h with different concentrations of TPW extracts: (**a**) HEK-293, (**b**) Hep-G2, (**c**) HeLa, and (**d**) HT-29. The **** symbol indicates significant differences detected among the tomato extracts and the positive control (H_2_O_2_) compared to the negative control, as determined by One-way ANOVA (*p* < 0.05).

The HEK-293 (normal cells), HEP-G2, HeLa, and HT-29 (tumoral cells) cell lines were used to assess the cytotoxic effects of TPW extracts at two concentrations (200 and 400 μg/mL) after 24 h of incubation. As shown in Figure 5a (HEK-293 cells), Figure 5b (HEP-G2 cells), Figure 5c (HeLa cells), and Figure 5d (HT-29 cells), there were significant concentration-dependent inhibitory effects of both TPWap and TPWas extracts. After treatment, the TPWap and TPWas extracts demonstrated a tumoral cell growth inhibitory effect compared to the control cells in HEP-G2, HeLa, and HT-29. Both doses were effective in all three cell lines. However, neither the TPWap nor TPWas extracts showed a growth inhibitory effect on HEK-293 (normal cells) compared to the control instead, all extracts exhibited a dose-dependent effect. Tumor cells seem to be more sensitive to TPW extracts compared with normal cells, the inhibition being under 50% in most of the samples.

**Figure 6:**
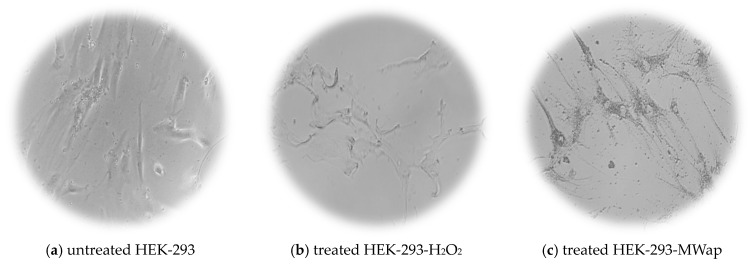

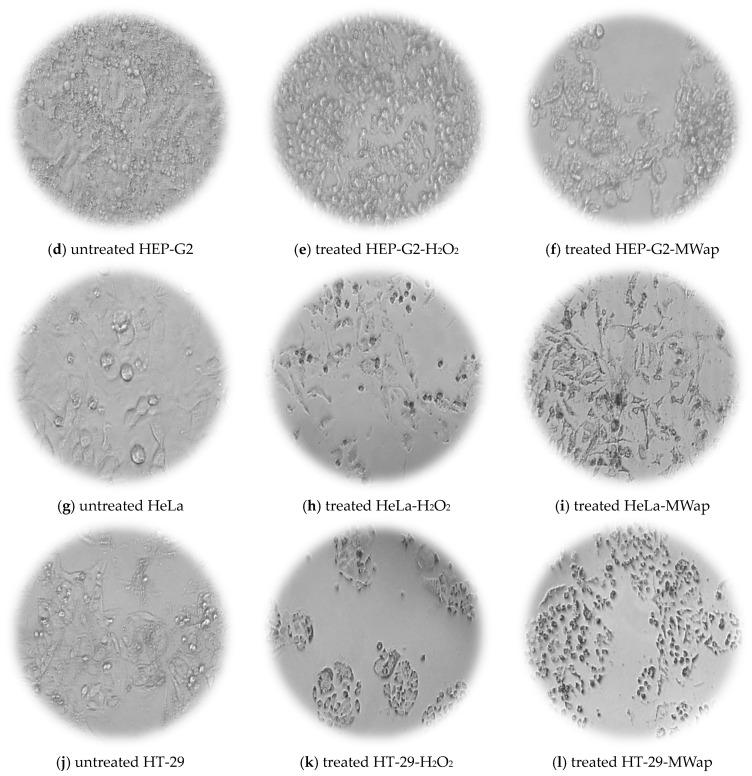
Cell morphology of (**a**,**d**,**g**,**j**) untreated cells (control negative) and (**b**,**e**,**h**,**k**) treated cells with H_2_O_2_ (control positive), and (**c**,**f**,**i**,**l**) treated cells with MWap extract.

The TPW extracts demonstrated a promising cytotoxic effect on HEP-G2, HeLa, and HT-29 tumoral cells, without affecting the HEK-293 normal cells. Figure 6 shows the morphological images of HEK-293 normal cells and HEP-G2, HeLa, and HT-29 tumoral cells: untreated cells (negative control) are shown in Figure 6a,d,g,j; cells treated with H_2_O_2_ (positive control) are in Figure 6b,e,h,k; and cells treated with MWap extract at 400 µg/mL are in Figure 6c,f,i,l. The images of MWap extract clearly display the induced morphological abnormalities in HEP-G2, HeLa, and HT29 tumoral cells, similar to the irregular cell shapes observed in the positive control, H_2_O_2_. However, the MWap extract causes some morphological abnormalities in HEK-293 normal cells. For the morphological presentation mentioned above, the MWap extract was selected to highlight its cytotoxic effect on tumor cells because it had the highest content of FOS, including total sugars, a high prebiotic-like substrate use in vitro effect, and antifungal effect, without exhibiting a cytotoxic effect on healthy cells.

## 4. Discussion

Building upon our previous research study on extraction optimization, which focused primarily on maximizing the yield of bioactive compounds from TPW [19], the present study extends these findings by evaluating the chemical composition, antifungal, prebiotic-like substrate uses in vitro, and cytotoxic effects of the TPW-optimized extracts. This approach offers new insights into the functional properties and potential applications of TPW extracts, extending beyond mere extraction efficiency to evaluate their biological relevance and practical utility.

The results clearly demonstrated the antifungal, prebiotic-like substrate use in vitro and cytotoxic effects of the TPW extracts. Tomato plants synthesize a range of health-promoting key phytochemical compounds, including vitamins, pigments, carbohydrates, fibers, minerals, flavonoids, phenolic acids, and glycoalkaloids [19,37]. Some of these compounds serve as part of their natural defense system and exhibit various biological effects, such as antibacterial, antifungal, antitumoral, and antiviral [38].

The GC-MS profile of the TPW extracts studied reveals a broad spectrum of volatile and semi-volatile compounds, within which variability was observed depending on the plant material analyzed and the extraction method applied. TPW extracts obtained through cascade extraction (CAS1 and CAS2) showed the highest number of compounds compared to the other two applied extraction methods (MAE and UAE). Each of the 16 identified compounds plays different roles and has varying importance, both in the physiological processes of the plant and in potential phytomedical applications [39,40,41]. For example, methyl esters of palmitic and linolenic acids, and phytol exhibit antioxidant and antimicrobial properties [42,43,44].

In addition to volatile and semi-volatile compounds, the results recorded for the TPW extracts confirm the presence of the O–H bonds in polysaccharides or phenolic compounds (3315 cm^−1^), and the presence of the C–O bonds in alcohol groups of soluble sugars or phenolic compounds (1115 cm^−1^ and 1022 cm^−1^). Vermeir et al. [45] also confirmed the presence of distinct molecular vibrations in soluble sugars of six tomato varieties, as evidenced by FTIR spectra. Furthermore, Animashaun and Sobowale [46] reported the presence of flavonoids in the two tomato varieties, as shown by FTIR spectra that featured a characteristic peak at 1640 cm^−1^, similar to that observed in the USas extract.

The soluble sugar analysis determined for the optimized TPW extracts was notably different from those reported in the literature for tomato fruits and other plant waste materials. The results demonstrate that the TPW extracts exhibit a significantly high FOS content. For instance, the FOS content reached values almost one order of magnitude higher than those described in tomato fruits by Anđelini et al. [47]. Although it can be stated that the TPWap extracts are richer in FOS, this aspect is strongly influenced by the tomato variety, climatic factors, as well as cultivation practices. Glucose concentrations were comparable to those reported in tomato pulp, while fructose levels were considerably lower, in agreement with previous findings. When compared with other plant waste matrices, such as tea leaves (processed green or black tea), apple leaves, and alfalfa [48,49,50], TPW extracts exhibited substantially higher levels of sucrose, glucose, and fructose, further underlining the efficiency of the applied extraction strategies.

Soluble sugars are well recognized as essential energy sources, produced directly by photosynthesis, and play a central role in plant metabolic pathways [51]. Sucrose is the principal soluble sugar involved in carbohydrate transport in plants and contributes to defense mechanisms. At the same time, its concentration strongly influences both the size and quality of tomato fruits [52]. Fructose has been reported to participate in defense processes at the leaf level. In contrast, glucose plays a dual role in leaf development and in serving as a key precursor in cellular energy metabolism [53]. According to the literature, MAE generally ensures higher yields of sugars and other bioactive compounds due to rapid heating and disruption of plant cell walls, while UAE promotes solubilization through cavitation and mechanical effects, which can favor the release of certain low-molecular-weight compounds. Studies such as those on alfalfa and garlic confirm this differential behavior [49,54]. Based on these findings, we expected MAE to enhance the recovery of polysaccharides and oligosaccharides, whereas UAE could favor the extraction of free sugars. The total soluble sugars identified vary significantly in the TPW extracts, depending on the extraction method and the type of plant material studied. Thus, MAE led to a higher content of FOS, sucrose, and glucose (MWap), whereas UAE resulted in a higher content of fructose (USas).

A total of 11 glycoalkaloids were identified in the TPW extracts, including tomatidine, dehydrotomatine, α-tomatine, acetoxytomatine, and seven α-tomatine derivatives. Glycoalkaloids are secondary metabolites found in the tomato plant. For instance, tomatine and dehydrotomatine are produced in all parts of the tomato plant [55,56]. A previous study [34] demonstrated the presence of glycoalkaloids in tomato leaves, predominance of α-tomatine and dehydrotomatine. Topolewska and Haliński [57] reported the total amount of glycoalkaloids in the tomato plant leaves of 12.5 ± 2.4 mg/g dry weight, the highest amount being for α-tomatine, followed by dehydrotomatine. Additionally, tomato plant glycoalkaloids are also known to provide a fungicidal effect [35].

Furthermore, the TPW extracts may also demonstrate significant antifungal effects, as they contain organic acids like acetic and oxalic acids, as well as phenolic compounds such as rutin, quercetin, kaempferol, and isorhamnetin glycosides, which have shown notable antifungal properties [58]. α-Tomatine exhibits amphipathic saponin properties that interact with sterol-containing microbial membranes, increasing permeability and leading to cell lysis or growth inhibition in sensitive microorganisms. TPW extracts exhibited inhibitory potential against *C. albicans* ATCC 10231, *C. parapsilosis* ATCC 22019, *C. glabrata* ATCC 64677, and *C. auris* 6328. A comparative analysis of the results indicates that the most potent antifungal effect was seen against *C. glabrata* ATCC 64677 for all TPW extracts, with the lowest MIC values. The sensitivity of different *Candida* species to tomato waste extracts could be explained by the membrane composition of the strains and the presence of efflux pumps. *C. glabrata* presents a higher membrane permeability and lower ergosterol content compared with *C. auris*. In other words, all tested extracts demonstrated antifungal activity to varying extents, with a clear distinction based on the type of plant material studied (axillary shoots or aerial parts mixture) and the extraction method used (MAE, UAE, CAS1, and CAS2). Compounds present in axillary shoots exert a higher antifungal effect on yeast strains compared with those present in aerial parts. In comparison, CAS1 and CAS2 extract methods exert a better effect than MAE and UAE in the case of *C. albicans* ATCC 10231. Furthermore, among the extracts of the “Bull’s Heart” tomato variety that were tested, the chloroform extract was the most active against *C. albicans*. In contrast, the ethyl acetate extract was an exception and showed no activity. Hexane extracts from both the “Bull’s Heart” and “Cherry” varieties were also active against *C. albicans* [59].

Sources of plants that include non-digestible short-chain carbohydrates frequently improve the function and effectiveness of certain beneficial microbiota in the human gut [60]. FOS are directly involved in modulating the intestinal microbiota and exerting prebiotic-like substrate utilization effects in vitro due to the ability of LAB strains to metabolize these compounds (i.e., enzymes such as fructokinase and sucrose-6-phosphate hydrolase) compared with other microorganisms [61]. The high FOS content identification in TPW extracts (*L. esculentum* hybrid Cheramy RZ F1) represents a novel aspect of this study, positioning them as a promising source of prebiotic-like substrate for in vitro carbohydrate use. A positive prebiotic effect is associated with the ability of the prebiotic substrate to be selectively metabolized only by probiotic microorganisms, and not by the remaining intestinal microbial strains, which thoroughly supports the growth of beneficial microorganisms [62].

Natural products are among the most successful sources of phytochemically active compounds for treating tumors in humans, based on evidence from long-term traditional use spanning multiple generations [63]. Different mechanisms have been identified that contribute to tumor prevention through the active compounds in tomatoes, including the inhibition of inflammatory processes and the disruption of tumor angiogenesis [64]. Consuming these active compounds directly seems to be more effective when taken in their natural biological matrix as dietary supplements [65]. Additionally, tomato plant glycoalkaloids have naturally occurring functional effects such as lipid-lowering, antioxidation, antiaging, and antitumoral [66]. Overall, the results suggest that treatment with TPWas and TPWap extracts can inhibit the growth effects in HEP-G2, HeLa, and HT-29 tumoral cell lines, with minimal impact on HEK-293 normal cell line. Results that have also been demonstrated in other previous studies on the same types of cell lines and gastric cell lines [67,68,69]. Also, glycoalkaloids, namely tomatidine and α-tomatine, present in the TPW extracts, showed high antitumoral activity against the prostate tumoral cells, PC3 [70], and liver tumoral cells, HEP-G2 [71]. The concentrations of glycoalkaloids were lower compared with the 200 mg/kg safety threshold for food. The potential toxicity can be mitigated by thermal or enzymatic treatment or by fermentation.

## 5. Conclusions

Tomato plant waste extracts (*L. esculentum* hybrid Cheramy RZ F1) reveal several key findings regarding the composition and potential of various plant-based alternatives. Firstly, tomato plant waste extracts stand out as the source of the highest amounts of carbohydrates and phenolic compounds among the waste alternatives assessed. Secondly, tomato plant waste extracts emerge as a promising candidate due to their significantly higher antifungal, prebiotic, and cytotoxic effects. These findings, taken together, support the potential of tomato plant waste extracts as a functional food ingredient for improving intestinal barrier function and modulating the gut microbiota while respecting the importance of food safety to mitigate risks associated with the key phytochemical compounds present in these extracts.

## Figures and Tables

**Table 1:** Comprehensive description of tomato plant extracts.

No.	Extract Code	Part of the Plant	Solvent (%)	Plant: Solvent Ratio	Time (Min)	Temperature (°C)	Extraction Method	Extraction Code
1	MWap	aerial parts mixture (ap)	78	39	60	79	microwave- assisted extraction	MAE
2	MWas	axillary shoots (as)	73	39	60	125		
3	USap	aerial parts mixture (ap)	70	36	60	56	ultrasound- assisted extraction	UAE
4	USas	axillary shoots (as)	65	40	60	70		
5	C1ap	aerial parts mixture (ap)	78	39	120	79	full-time cascade extraction	CAS1
6	C1as	axillary shoots (as)	73	39	120	125		
7	C2ap	aerial parts mixture (ap)	78	39	70	79	short-time cascade extraction	CAS2
8	C2as	axillary shoots (as)	73	39	70	125		

**Table 2:** Volatile and semi-volatile compounds present in TPW extracts.

RT (min)	Compound Name	RRI	MW	Area (%)
19.98	Hexyl-methylcyclopentane	1291	168.32	MWap—0.18; USap—1.21; C2ap—1.10
26.69	3′,5′-Dimethoxyacetophenone	1567	180.20	USap—1.52
34.43	Hexadecatrienoic acid methyl ester	1904	264.41	USap—0.71; USas—1.96
35.06	Palmitic acid, methyl ester	1934	270.46	MWap—0.79; MWas—16.05; USap—9.39; USas—1.79; C1ap—11.02; C1as—15.77; C2ap—12.83; C2as—13.52
38.44	Octadecadienoic acid, methyl ester	2101	294.48	MWap—0.57; MWas—20.62; USap—6.73; USas—1.78; C1ap—8.76; C1as—19.52; C2ap—9.51; C2as—16.08
38.54	Octadecatrienoic acid, methyl ester	2107	292.46	MWap—1.02; MWas—22.35; USap—13.27; C1ap—15.94; C1as—22.23; C2ap—17.47; C2as—18.80
38.80	Phytol	2119	296.54	MWap—0.90; MWas—13.46; USap—29.70; USas—23.66; C1ap—14.10; C1as—12.77; C2ap—19.89; C2as—16.97
39.07	Methyl stearate	2135	298.51	MWap—0.33; MWas—4.39; USap—3.81; USas -1.94; C1ap—3.05; C1as—4.43; C2ap—4.19; C2as—4.22
39.99	Octadecatrienol	2175	264.45	USap—3.88; USas—4.55
40.00	Octadecanamide	2185	283.50	MWap—0.15; MWas—0.92; C1ap—0.98; C1as—1.15; C2ap—1.94; C2as—1.58
42.79	Methyl 18-methylnonadecanoate	2336	326.56	MWas—1.21
43.22	Linoleic acid ethyl ester	2361	306.49	MWas—1.39
43.30	Octadecenamide (Oleamide)	2367	281.48	MWas—7.59; USap—17.14; USas—29.73; C1ap—8.14; C1as—8.43; C2ap—13.79; C2as—12.10
43.70	Nonadecanamide	2393	297.52	C2ap—2.16
45.52	Cyclopropaneoctanoic acid, 2-[[2-[(2-ethylcyclopropyl)methyl]cyclopropyl]methyl]-, methyl ester	2476	334.54	MWas—2.00; USap—3.45; USas—5.90
52.04	2-Methylhexacosane	2708	380.74	MWas—0.86; C2as—1.24

**Table 3:** Soluble sugars present in TPW extracts.

Extract Code	Soluble Sugars (mg/g d.e.)				Total (mg/g d.e.)
	Sucrose	Glucose	Fructose	Inulin (FOS)	
MWap	168 ± 1 ^a^	101 ± 2 ^a^	21 ± 1 ^b^	499 ± 6 ^a^	788
MWas	44 ± 1 ^e^	59 ± 1 ^c^	12 ± 0 ^e^	345 ± 3 ^d^	460
USap	114 ± 3 ^bc^	73 ± 0 ^b^	18 ± 1 ^c^	372 ± 14 ^c^	577
USas	45 ± 1 ^e^	50 ± 1 ^d^	26 ± 1 ^a^	377 ± 8 ^bc^	497
C1ap	111 ± 7 ^c^	71 ± 2 ^b^	16 ± 1 ^d^	374 ± 5 ^c^	573
C1as	42 ± 2 ^e^	37 ± 0 ^f^	11 ± 0 ^e^	327 ± 7 ^e^	417
C2ap	119 ± 6 ^b^	74 ± 3 ^b^	16 ± 1 ^d^	392 ± 6 ^b^	601
C2as	56 ± 2 ^d^	46 ± 1 ^e^	12 ± 1 ^e^	334 ± 4 ^de^	448

d.e.—dried extract. Superscript letters within the same column indicate statistically significant differences among the samples, as determined by ANOVA (*p* < 0.05); data are presented as mean ± standard deviation.

**Table 4:** Glycoalkaloids present in TPW extracts.

Alkaloid Name	Molecular Formula	[M+H]^+^ (*m*/*z*)	MS/MS Fragments	References
Tomatidine derivative	-	528.7716	416.3530, 257.1902, 122.0963	[31]
α-Tomatine derivative 1	C_39_H_65_NO_12_	743.2025	695.3648, 541.2615, 427.1108, 348.1867	-
Dehydrotomatine	C5_0_H_81_NO_21_	1032.5406	922.0105, 755.4210, 576.3910, 414.3375, 246.1136, 163.0420	[32,33]
α-Tomatine	C_50_H_83_NO_21_	1034.5562	814.2947, 578.4061, 416.3541, 295.1034, 122.0964	PubChem, [33,34]
α-Tomatine derivative 2	C_50_H_85_NO_22_	1051.6212	855.5202, 537.3059, 353.2707, 279.2338, 122.0977	-
Acetoxytomatine	C_52_H_85_NO_23_	1092.5629	1032.5408, 864.4839, 636.4057 557.7672, 414.3281, 295.0930	[35]
α-Tomatine derivative 3	-	1197.5930	1034.5558, 960.5167, 578.4049, 416.3501, 295.1016	-
α-Tomatine derivative 4	-	1237.5882	1050.5525, 707.2919, 594.3958, 432.3422, 295.0965, 122.0893	-
α-Tomatine derivative 5	-	1292.6559	1034.5505, 922.0098, 662.4241, 527.7613, 295.0993, 122.0932	-
α-Tomatine derivative 6	-	1363.6905	1050.5518, 919.4515, 594.3924, 314.1272, 177.0432	-
α-Tomatine derivative 7	-	1367.6250	1034.5587, 871.4687, 709.4112, 578.3980, 416.3424, 295.0912	-

**Table 5:** Antifungal effect of TPW extracts against *Candida* species.

*Candida* Species	TPW Extract							
	MWap	MWas	USap	USas	C1ap	C1as	C2ap	C2as
*C. albicans* ATCC 10231	+	++	+	+++	+	++	+	++
*C. albicans* CMGBy 18	+	+	+	+	+	+	+	+
*C. parapsilosis* ATCC 22019	+/−	++	+/−	++	+/−	+	+/−	++
*C. glabrata* ATCC 64677	+++	+++	++	+++	+++	+++	++	+++
*C. auris* DSM 21092	+/−	+/−	+/−	+/−	+/−	+/−	+/−	+/−
*C. auris* 6328	+/−	+/−	+/−	+/−	+/−	+/−	+/−	+/−

+/− lower antifungal activity; + moderate antifungal activity; ++ high antifungal activity; +++ higher antifungal activity.

**Table 6:** MICs of TPW extracts against *Candida* species.

*Candida* Species	TPW Extract (µg/mL)								FLU (µg/mL)
	MWap	MWas	USap	USas	C1ap	C1as	C2ap	C2as	
*C. albicans* ATCC 10231	1000	250	1000	250	500	250	500	250	15
*C. parapsilosis* ATCC 22019	1000	250	1000	250	1000	500	1000	250	31
*C. glabrata* ATCC 64677	125	125	250	125	125	125	250	125	50
*C. auris* 6328	1000	1000	1000	1000	1000	1000	1000	1000	<100

FLU—fluconazole.

## Data Availability

The original contributions presented in this study are included in the article. Further inquiries can be directed to the corresponding authors.
